# Health Extension Workers’ diagnostic accuracy for common childhood illnesses in four regions of Ethiopia: a cross-sectional study

**DOI:** 10.1111/apa.14888

**Published:** 2019-06-04

**Authors:** Theodros Getachew, Solomon Mekonnen, Mezgebu Yitayal, Lars Åke Persson, Della Berhanu

**Affiliations:** 1 Health System and Reproductive Health Research Directorate, Ethiopian Public Health Institute, Addis Ababa, Ethiopia; 2 College of Medicine and Health Science, Institute of Public Health, University of Gondar, Gondar, Ethiopia; 3 London School of Hygiene and Tropical Medicine, Bloomsbury, London, UK

**Keywords:** Childhood illnesses, Health Extension Worker, Community Health Worker, technology adoption, Integrated community case management, Quality of care

## Abstract

**Aim:**

The Ethiopian primary care of sick children is provided within the integrated Community Case Management of childhood illnesses by Health Extension Workers (HEW). There is limited knowledge whether this cadre correctly assess and classify common diseases. The aim was to study their ability to correctly classify common childhood illnesses.

**Methods:**

A survey was conducted from December 2016 to February 2017 in four regions of Ethiopia. Observations of the HEWs‘ assessment and classification of sick children were followed by child re-examination by a trained health officer.

**Results:**

The classification by the HEWs of 620 sick children as compared to the reexaminer had a sensitivity of 89% and specificity of 94% for diarrhoea, sensitivity 52% and specificity 91% for febrile disorders, and a sensitivity of 59% and specificity of 94% for acute respiratory tract infection. Malnutrition and ear infection had a sensitivity of 39 and 61%, and a specificity of 99 and 99%, respectively.

**Conclusion:**

Most cases of diarrhoea were correctly classified, while other illnesses were not frequently identified. The identification of malnutrition was especially at fault. These findings suggest that a significant number of sick children were undiagnosed that could lead to absent or incorrect management and treatment.

**Abbreviations**
HEP: Health Extension Program
HEWs: Health Extension Workers
iCCM: Integrated Community Case Management
OHEP: Optimization of the Health Extension Program
PHC: Primary Health Care
SNNP: Southern Nations, Nationalities and People
UNICEF: United Nations Children’s Fund
WHO: World Health Organization

## INTRODUCTION

The global under-five mortality rate has dropped from 69 deaths per 1000 live births in 2000 to 38 in 2016 (1). Even in Sub-Saharan Africa, the region with the highest under-five mortality rate, there was a substantial reduction (1). Ethiopia met the Millennium Development Goal 4 by reducing the under-five mortality from 205 deaths per 1000 live births in 1990 to 64 deaths per 1000 live births in 2013 (2).

The success of reducing the under-five mortality by two-thirds over the past 20 years could be attributed to improvements in multiple sectors including increased access to lifesaving health system interventions (2). These interventions include improved availability of outpatient care for sick children, expanded coverage of immunisations and an increased number of facilities that provide treatment, growth monitoring and nutrition services (3).

Despite this improvement, the under-five mortality in Ethiopia remains high. The significant causes of death in Ethiopia are, according to national-level statistics, lower respiratory tract infections, diarrhoeal diseases and neonatal conditions, such as preterm birth, birth complications, asphyxia and neonatal sepsis (4). Malnutrition is a major contributing cause to the under-five deaths (4).

Key notes

- There is insufficient knowledge whether Health Extension Workers (HEWs) accurately assess and classify childhood illnesses.
- The results of this study imply that a significant number of sick children were not correctly diagnosed, which could lead to absent or incorrect treatment.
- Efforts are needed to improve the quality of HEWs’ diagnostic ability for childhood illnesses and their adherence to the guidelines for the examination, classification and treatment of childhood illnesses.

The World Health Organization (WHO) and the United Nations Children’s Fund (UNICEF) have suggested an integrated Community Case Management (iCCM) of common childhood illnesses as an essential strategy to foster equitable services and to contribute to a sustained reduction of child mortality (5). The approach includes interventions to improve the skills of health workers, the health system, and family and community practices (6). There is also evidence that iCCM can enhance the quality of care provided by the health workers (7). It has been shown that children within the iCCM programme receive better quality of care (8).

Ethiopia introduced the iCCM programme in 2010 as part of the national Health Extension Program (HEP). The HEP was launched by the Federal Ministry of Health in 2003 (9) and has been implemented at the community level by Health Extension Workers (HEWs) at the health posts. The health posts are equipped with basic equipment and pharmaceutical drugs such as thermometers, measuring tapes, rapid tests for malaria, scales, growth monitoring charts, antibiotics and antimalarial drugs.

There were, however, concerns that the utilisation of iCCM services was low (10). The reasons included absent HEWs at the health posts, little trust in the HEWs (11), perceptions of which illness severity they could manage, the quality of their services (12), lack of community awareness of services provided at the health post (13), limited skills of HEWs in the management of sick children and lack of drugs and other supplies (14).

Based on these gaps, the Optimization of the Health Extension Program (OHEP) intervention was initiated in 2016 by the Federal Ministry of Health, UNICEF and PATH to increase the utilisation of high-quality iCCM and neonatal health services. This intervention included three strategies: community mobilisation, capacity building for the primary care workers to provide high-quality services and efforts to improve health system ownership and accountability of iCCM and neonatal health services. The present study was part of the baseline survey that included household and health facility assessments as a first step in the OHEP evaluation.

Given the conflicting evidence on the quality of services provided by the HEWs within the iCCM programme, this study aimed to assess their ability to correctly classify common childhood illnesses in a sample of sick children from four regions of Ethiopia.

## METHODS

### Study design and area

A cross-sectional survey was conducted in four regions (Amhara, Southern Nations, Nationalities and People [SNNP], Oromia, and Tigray) of Ethiopia from December 2016 to February 2017.

Participants The study population was children with some illness aged 2–59 months, who were mobilised to visit health posts in the study areas. A total of 200 enumeration areas had been selected to represent the districts in the four regions. An enumeration area had an average household number of 150–200. Health posts serving these areas were included in the survey. In each health post’s catchment area, mobilisation was done to recruit sick children to seek care at the health post. The study aimed to assess four children, who were considered ill by their caretakers, brought to the health post for assessment, illness classification and management by the HEW. This assessment by the HEW was followed by re-examination of the child by a health officer in the study team. A sample size of 800 sick children was considered to have 80% power to detect a difference of at least 15 percentage points when comparing the ability of HEWs with re-examiners (regarded as ‘gold standard’) to correctly classify diseases.

### Data collection

There were 15 data collection teams. Each team included an observer of the HEW‘s examination of the child and a health officer who re-examined the child. The observers were health officers or nurses, while the re-examiners were health officers with training in the Integrated Management of Childhood Illnesses and iCCM before recruitment.

After recruitment, data collectors were trained for two weeks covering study procedures, questionnaires, data collection techniques, clinical guidelines, quality-assurance procedures and study ethics. In addition, the re-examiners got refresher training in iCCM. They were also provided with a field manual.

### Data collection tools

The survey tools were based on previous WHO health facility survey tools that had been pilot-tested and customised to the local context. The assessment tool comprised of an observation of the HEW‘s assessment and classification of the sick child and a re-examination of the child by a health officer. The health officer re-examined the ill child and classified the illness according to the iCCM guidelines.

### Measurements

A computer-assisted field editing approach with computer tablets was used to collect the data. The observation and re-examination were registered on paper questionnaires and after that entered to the tablets in the field. Field teams were instructed to correct any identified errors or inconsistencies during the data collection. All data entry and editing programmes were written using the Census and Survey Processing software. Statistical analysis was done using STATA v14.1 (Stata Corp LP, College Station, TX, USA). Socio-demographic characteristics of the sick children were described. The HEW‘s and the re-examiners’ classifications were cross-tabulated to compute sensitivity, specificity, predictive values and kappa statistics (15). Sensitivity was defined as the proportion of true positives that were correctly identified by the HEWs’ classifications (16). Specificity was the proportion of true negatives that were correctly identified by the HEWs’ classifications (16). Kappa measures the level of agreement beyond what was not due to chance (17). The kappa statistic is scaled to be zero when the amount of agreement expected to be observed is by chance and one when there is perfect agreement. For intermediate values, Landis and Koch (18) suggested the following interpretations: 0.0 poor; 0.00–0.20 slight; 0.21–0.40 fair; 0.41–0.60 moderate; 0.61–0.80 substantial; and 0.81–1.00 almost perfect agreement. The positive and negative predictive values are the proportions of positive and negative results in the HEW‘s classifications that are truly positive and negative results, respectively.

### Definitions of illness classification

The HEWs and the re-examiners were expected to classify acute respiratory tract infection, diarrhoea or dysentery, malnutrition, and ear infection based on the iCCM chart booklet (6). The chart booklet shows the sequence of steps in performing the clinical algorithms to assess, classify and treat sick children. The sick children were then classified as having or not having any of the major childhood illnesses. Disease classification from the HEWs’ and the re-examiners was compared and analysed as to agreement in the classification of illnesses.

### Ethical considerations

The Ethical Review Boards of the Ethiopian Public Health Institute (protocol number SERO-012-8-2016; Version 001, August 2016), the London School of Hygiene and Tropical Medicine (protocol number 11235), June 2016, and the University of Gondar (protocol number O/V/P/RCS/05/371/2018), December 2018, reviewed and approved the study. An information sheet of the survey was translated into the local languages Amharic, Oromiffaa, and Tigrigna and read to caregivers to get informed consent. Confidentiality of all study participants was assured; no personal identifiers were included. Participants with an acute illness that could not be managed at the health post were referred to a higher health facility level. Re-examiners informed the HEWs of any missing diagnoses in need of treatment for immediate additional action.

## RESULTS

### Participation

A total of 800 sick child consultations were expected to be observed at 200 health posts. Twenty-five of the enumeration areas shared a health post. Six health posts from the SNNP region in Konso district were excluded due to local unrest. This study covered a total of 147 health posts, indicating that 22 health posts did not have any children mobilised. An average of four sick children was seen per health posts, and it ranged from one up to eight sick children per health post. Almost all HEWs were women; only four out of the 186 HEWs were men. On average, nine out of every ten HEWs had been trained in the iCCM of childhood illnesses, and half had received this training during the past four years.

We observed and re-examined a total of 620 sick children from the four regions (Table 1). A majority of the children were from the Amhara and Oromia regions. One-third were in the age group 2–11 months and a similar proportion in the interval 12–23 months. Among the examined children, a bit more than half were boys. Among the reasons to bring the children to health posts, respiratory problems, diarrhoea and fever dominated. Only one caretaker stated malaria as the reason to attend the health post. Among the other reasons presented, pain in various parts of the body dominated, especially abdominal pain.

**Table 1:** Characteristics of sick under-five children at health posts that were offered integrated Community Case Management services in four regions of Ethiopia, December 2016 to February 2017

Characteristics	N = 620	
	Frequency	Per cent (95% confidence interval)
Region		
Amhara	265	43 (39–47)
Oromia	197	32 (28–36)
Tigray	90	15 (12–18)
Southern Nations, Nationalities, and People	68	11 (9–14)
Age		
2–11 months	203	33 (29–37)
12–23 months	190	31 (27–34)
24–35 months	95	15 (13–18)
36–47 months	75	12 (10–15)
48–59 months	57	9 (7–12)
Sex		
Boys	337	54 (50–58)
Girls	283	46 (42–50)
Complaints presented as the reason to seek care at the health post		
Cough or difficult breathing	360	58 (55–61)
Diarrhoea	224	36 (33–39)
Vomiting	84	14 (11–16)
Fever	163	26 (24–29)
Ear problem	37	6 (4–7)
Other	69	11 (9–13)

### Validity of disease classification by HEWs

The cross-tabulation of illness classification by HEWs and re-examiners is presented in Table 2. The HEW and reexaminer had an agreement in the assessment of whether the illness was present or not in 83% of fever or malaria classifications, and 88% of acute respiratory tract infection classifications. Similarly, the HEWs and the re-examiners agreed in 94% of diarrhoea and malnutrition, and in 97% of ear infection classifications.

**Table 2:** Level of agreement between the Health Extension Workers (HEW) and the health officers (observation and re-examination) classification of childhood illnesses in four regions of Ethiopia, December 2016 to February 2017

Childhood illnesses classification by HEWs	Childhood illnesses classification by re-examination					
	Yes		No		Total	
	n	%	n	%	n	%
Fever or malaria						
Yes	69	11.1	43	6.9	112	18.1
No	64	10.3	443	71.6	507	81.9
Total	133	21.5	486	78.5	619	100.0
Acute respiratory tract infection						
Yes	63	10.2	30	4.8	93	15.0
No	44	7.1	482	77.8	526	85.0
Total	107	17.3	512	82.7	619	100.0
Diarrhoea or dysentery						
Yes	210	33.9	12	1.9	222	35.9
No	25	4.0	372	60.1	397	64.1
Total	235	37.9	384	62.0	619	100.0
Malnutrition						
Yes	26	4.2	5	0.8	31	5.0
No	41	6.6	546	88.3	587	95.0
Total	67	10.8	551	89.1	618	100.0
Ear infection						
Yes	25	4.1	3	0.5	28	4.6
No	16	2.6	570	92.8	586	95.4
Total	41	6.7	573	93.3	614	100.0
Childhood illness						
Yes	350	58.1	144	23.9	494	82.1
No	73	12.1	35	5.8	108	17.9
Total	423	70.2	179	29.7	602	100.0

The ability of the HEWs to correctly identify those with one of the common childhood illnesses is presented in Table 3. Overall, low sensitivity and relatively high specificity were observed. The sensitivity for malnutrition was particularly low, but the ability to truly identify children with the illness was even low for fever, acute respiratory tract infection and ear infections. The Kappa statistics ranged from 0.46 to 0.87.

**Table 3:** Kappa statistics, sensitivity, specificity and predictive values of Health Extension Workers’ assessment and classification of sick children based on direct observation and reexamination study at health posts in four regions of Ethiopia. December 2016-February 2017

Childhood illnesses	Kappa coefficient	Sensitivity (95% CI)	Specificity (95% CI)	Positive predictive value (95% CI)	Negative predictive value (95% CI)
Fever or malaria	0.46	51.9 (43.1–60.6)	91.2 (88.3–93.5)	61.6 (53.6–69.0)	87.4 (85.3–89.2)
Acute respiratory tract infection	0.56	58.9 (49.0–68.3)	94.1 (91.7–96.0)	67.7 (58.9–75.5)	91.6 (89.7–93.2)
Diarrhoea or dysentery	0.87	89.4 (84.7–93.0)	96.9 (94.6–98.4)	94.5 (90.9–96.8)	93.7 (91.1–95.6)
Malnutrition	0.50	38.8 (27.1–51.50)	99.1 (98.0–99.7)	83.9 (67.4–93.0)	93.0 (91.7–94.2)
Ear infection	0.71	61.0 (45.0–76.0)	99.5 (98.5–99.9)	89.3 (72.4–96.4)	97.3 (96.1–98.1)

**Figure 1:**
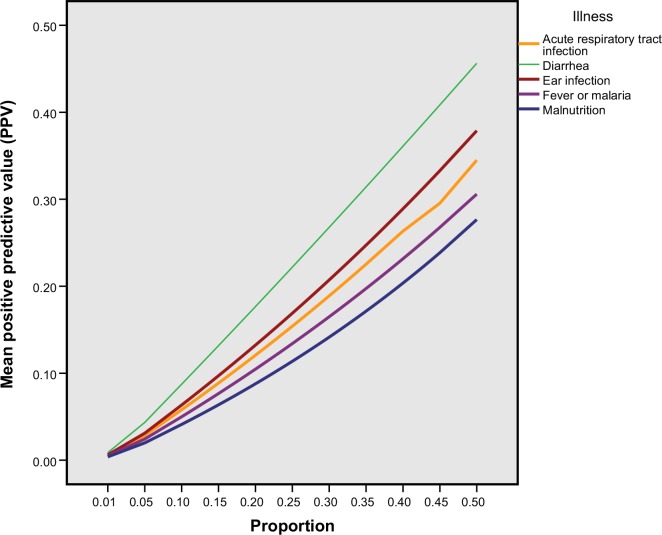
Calculated positive predictive values of the Health Extension Workers’ assessments of different illnesses with sensitivity varying from 38 to 89% and specificity from 89 to 99.5% if disease proportions are in the range 0–50%. Health post survey in four regions of Ethiopia, December 2016 to February 2017

The consequences of these levels of sensitivity and specificity for the ability to correctly identify an individual (positive predictive value) are displayed in Figure 1. In the screening process, the predictive value varies with the varying prevalence of different childhood illnesses. As illustrated in the figure, when the proportion for all childhood illnesses in the population increase, the probability of having the actual disease among a child classified to have the diseased also increase, although still on a very low level.

Table 4 shows the assessment, classification and treatment by the HEWs of children presenting with diarrhoea or respiratory symptoms at the health post. There were 360 children having a cough or rapid or difficult breathing. For one-quarter of the children with such complaints, the respiratory rate was not counted. Among children with respiratory symptoms, 24% were assessed as fast breathing rate. Thirty-one per cent of the children classified to have suspected pneumonia were treated with antibiotics.

**Table 4:** Assessment, classification and treatment by the Health Extension Workers of children presenting with diarrhoea or respiratory symptoms at health posts in four regions, Ethiopia. December 2016-February 2017

**Children presenting with respiratory symptoms complaint (N = 360)**	**%(95% CI)**
Assessment	
Not assessed including counting respiratory rate	26 (22–29)
Assessed as fast breathing rate	24 (21–28)
Assessed as normal breathing rate	50 (46–54)
Classification	
Classified as acute respiratory tract infection	26 (23–29)
Treatment	
Treated with antibiotics	31 (27–34)
**Children presenting with diarrhoea complaints (N = 224)**	**% (95% CI)**
Diarrhoea confirmed in assessment	94 (91–97)
Assessed for dehydration	32 (27–37)
Classified as dehydrated	14 (10–17)
Treated with oral rehydration solution	60 (55–64)

Diarrhoea was the second most common presenting symptom of children attending the health post. Overall, caretakers of 224 children reported such complaints. One-third was assessed for dehydration. Fourteen per cent of those were classified as dehydrated, and 60% received Oral Rehydration Solution.

## DISCUSSION

This study has shown that Ethiopian HEWs from four regions had low sensitivity for children with malnutrition, febrile illnesses or acute respiratory tract infection, while diarrhoea cases had high sensitivity.

Only 6 out of 10 children with respiratory tract infection were correctly identified. These deficiencies might be a result of errors in performing assessments. The HEWs did not count the respiratory rate in a quarter of the sick children despite caregivers’ complaints of respiratory problems. In a study conducted with Community Health Workers in Zambia, the sensitivity in the screening for respiratory infections was 81% (19). In that study, the health workers were directly observed with measurement of the respiratory rate of children with suspected pneumonia recorded by video to compare against a reference.

The kappa coefficient of agreement showed moderate agreement for respiratory tract infections in our study, which is also similar to results from a survey conducted in India among nurses (20), who were recruited from a Neonatal Intensive Care Unit.

In our study, only 4 out of 10 children with malnutrition were correctly identified. This result was most likely due to errors in performing assessments. Two-thirds missed assessing dehydration when having diarrhoea. Theoretically, the re-examiners might also have over-diagnosed children with malnutrition. The re-examiners were, however, health officers who had received training in the assessment of malnutrition and followed the iCCM guidelines in their examination of the child. Health officers, the highest level of cadre to implement iCCM, are clinicians who had performed promotive, preventive, curative and rehabilitative services including management and implementation of Primary Health Care services compatible to the needs of the population.

In our study, nine out of ten children with diarrhoea or dysentery were correctly diagnosed by HEWs. The results from a study with voluntary health workers conducted in Uganda also revealed that the assessment and classification of diarrhoeal diseases were done with better accuracy than for other illnesses (21).

The HEWs correctly classified only half of the children with fever. There was only one case of malaria identified by the re-examiners using rapid diagnostic tests. Several of the study areas were situated at a high altitude, and for the lower altitude areas, malaria transmission was relatively low, due to the season of the year. This study did not aim at separating fever due to malaria from fever due to other reasons. The low validity of the HEWs’ classification of febrile conditions might be a result of errors both in performing clinical assessments and in interpreting the findings and classifying the illness (22). A previous Ethiopian study, however, showed that, given that the diagnosis was correct, the HEWs provided high-quality care to sick children (7).

Due to the different treatment algorithms for zero to two months and the difficulty of mobilising newborns, this study focused on 2–59 months old children. The small number of children with some symptoms and signs limited our ability to evaluate the HEWs’ ability to classify children with anaemia and epidemic diseases such as measles. Evidence suggests that when the health care providers are aware of being observed about their performance, they alter their practices, the phenomenon that is known as the ‘Hawthorne effect’ (23). Direct observation of a health worker examining a sick child followed by re-examination is, however, a standard method to evaluate the clinical performance of health workers at health facilities (24). In spite of this potential bias, a considerable proportion of sick children with common childhood illness were not appropriately classified based on their symptoms. As such, our findings differ from previous results that suggest that direct observation could overestimate the performance of HEWs (25).

The sampling of this survey was not done to represent the four regions; the selection of districts was based on the planned interventions. Still, it is plausible that the results are typical for these selected districts and regions.

The goal of diagnosing children is to reduce childhood morbidity or mortality from illnesses by detecting the illnesses at an early stage to provide treatment successfully. If it fails to do so, a substantial number of children will remain undiagnosed, potentially leading to a lack of treatment and risk of death. The sensitivity is usually given more weight than specificity at the primary care level. A child is likely to suffer more from the lack of treatment, even if unnecessary use of therapy, especially antibiotics, increase the risk of resistance problems (26). Predictive values are influenced by the prevalence of the disease in the population that is being examined. If we examine sick children when the prevalence of an illness is high, it is more likely that the child, who is classified as having the disease by the HEWs, indeed has the disease.

These problems in the HEWs’ assessment and classification of sick children could be improved by further capacity building of HEWs, which could include training and supportive supervision and performance review meetings to increase their confidence and skill in the assessment, classification and management of sick children (27). It is also good to create increased demand for high-quality services and improve the health system ownership of the iCCM programme by optimising the HEP through community mobilisation, capacity building, and health system ownership and accountability.

A lot of reasons could result in the low performance of the HEWs in diagnosing under-five children. A meta-review of interventions to improve quality of care identified the following barriers to improved quality: communication barriers, lack of accountability, a variation of guidelines, shortage of resources and lack of studies assessing the role of leadership in improving quality of care (28).

The working conditions may also be essential factors for the improvement of the quality of services provided by the HEWs. There is growing evidence that the health system context matters for the quality of the services provided (29). There is a need to go beyond measuring the ‘hardware’ of the health system to capturing the ‘software’, that is contextual issues (30).

## CONCLUSION

The Ethiopian HEWs’ assessment and classification of sick children 2–59 months of age was validated by a re-examination performed by trained health officers. Most cases of diarrhoea were correctly classified, while fever and respiratory infections frequently were not identified. The identification of malnutrition was especially missed. These results suggest that a significant number of sick children were undiagnosed that could lead to absent or incorrect management and treatment. Efforts are needed to improve the quality of the diagnosis and classification of childhood illnesses done by the HEWs. Emphasis should be given to enhancing the ability of HEWs to adhere to the iCCM guidelines.

## Data Availability

The data for this manuscript were primarily collected by the Ethiopian Public Health Institute (EPHI) and London School of Hygiene and Tropical Medicine. Interested researchers may contact the focal person, Dr. Della Berhanu, at the EPHI, Addis Ababa, Ethiopia, through email; della.berhanu@lshtm.ac.uk.
